# Behavioral Fever in Lined Seahorse (*Hippocampus erectu)* Enhances the Immune Response to *Vibrio harveyi* Infection

**DOI:** 10.3390/ani15111509

**Published:** 2025-05-22

**Authors:** Siping Li, Xin Liu, Tingting Lin, Dong Zhang

**Affiliations:** 1 Key Laboratory of Inland Saline-Alkaline Aquaculture, Ministry of Agriculture and Rural Affairs, East China Sea Fisheries Research Institute, Chinese Academy of Fishery Sciences, Shanghai 200090, China; lisiping@ecsf.ac.cn (S.L.); liux@ecsf.ac.cn (X.L.); 2 Shangdong Qianyuan Bio-Tech Co., Ltd., Bingzhou 256600, China

**Keywords:** lined seahorse, behavioral fever, thermoregulation, immune response, temperature

## Abstract

Ectotherms can elevate their body temperature in response to infection by seeking warmer environments, a phenomenon known as behavioral fever. This adaptive response, widely documented in fish, activates immune defenses and improves survival. To explore an eco-friendly approach for managing Vibrio-induced enteritis in lined seahorse (*Hippocampus erectus*) aquaculture, we investigated whether *Vibrio harveyi* infection triggers behavioral fever and enhances immune function. Seahorses were intraperitoneally injected with *V. harveyi* (1 × 10^7^ cfu/fish) and placed in a thermal gradient tank (19–31 °C), allowing free movement between chambers. Challenged seahorses exhibited a significant preference (*p* < 0.05, 1.31-fold) for warmer zones compared to unchallenged controls, whereas no such difference (*p* > 0.05) was observed in a constant temperature (25 °C) tank, confirming behavioral fever. Furthermore, fevered seahorses showed significantly elevated plasma cytokine levels (PGE2, IL-1β, IL-6, and TNF-α; *p* < 0.05), which normalized (*p* > 0.05) to baseline levels, except for TNF-α, compared to unfevered individuals. In kidney tissue, challenged seahorses expressing behavioral fever exhibited gene expression levels (*tnf-α*, *il-6*, *ifn-g*, and *il-10*) similar to unchallenged controls (*p* > 0.05) but significantly lower (*p* < 0.05) than those kept at constant temperature. These findings suggest that behavioral fever in *H. erectus* modulates core temperature to regulate cytokine release and immune-related gene expression. This study provides foundational insights for developing practical, non-invasive strategies to mitigate enteritis in seahorse aquaculture through thermal behavior manipulation.

## Simple Summary

Behavioral fever, a thermoregulatory strategy where ectotherms migrate to warmer environments to enhance immune and survival, is well documented in many fish species. However, its existence in the lined seahorse (*Hippocampus erectus*) remains unexplored. In this study, we observed that *Vibrio harveyi*-infected seahorses consistently preferred warmer zones (31 °C) in a temperature gradient tank (19–31 °C), a behavior absent in uninfected individuals. Infected seahorses exhibiting fever displayed elevated plasma cytokine levels and downregulated immune-related gene expression patterns resembling those of uninfected controls. Our findings highlight the potential of manipulating thermal environments in seahorse aquaculture to activate behavioral fever, thereby improving disease resistance without relying on antibiotics.

## 1. Introduction

Unlike endotherms, which regulate body temperature through both physiological and behavioral mechanisms, ectotherms such as fish rely primarily on behavioral thermoregulation, namely seeking warmer environments to elevate their core temperature outside their optimal thermal range [1]. When infected by pathogens, ectotherms often exhibit behavioral fever, a response characterized by active movement toward warmer microhabitats to raise body temperature [1,2,3]. First documented in teleost fish (e.g., *Lepomis macrochirus* and *Micropterus salmoides*) by Reynolds et al. [4], behavioral fever has since been observed in diverse species. For instance, *Oreochromis niloticus* infected with *Edwardsiella piscicida* migrated from 28 °C to 34 °C [5]; *Cyprinus carpio* migrated from 28 °C to 32 °C in response to Cyprinid herpesvirus 3 (CyHV-3) infection [3]; Infectious Pancreatic Necrosis virus (IPNV)-treated *Salmo salar* elevate body temperature by 3–5 °C [6,7], and synthetic dsRNA-injected *Danio rerio* observed in the form of a mean 3 °C shift in thermal preference maintained over a 24 h period [8]. Behavioral fever shares evolutionary parallels with mammalian fever, as exogenous pyrogens trigger immune cells to release key pyrogenic mediators (e.g., prostaglandin E2 PGE2), ultimately inducing fever [8,9,10,11,12,13,14].

This thermoregulatory behavior is crucial for mounting effective immune responses in aquatic animals. By selecting warmer thermal environments, fish can modulate their immune responses, enhancing lymphocyte proliferation, inflammatory cytokine release, and other immune functions [15,16]. For instance, rainbow trout (*Oncorhynchus mykiss*) injected with bacterial lipopolysaccharide (LPS) shifted their thermal preference from 14.9 °C to 16.0 °C, resulting in significantly elevated IL-1β expression in the head kidney compared to fish maintained at a constant 13.5 °C [17]. Similarly, Atlantic salmon (*Salmo salar*) infected with infectious pancreatic necrosis virus (IPNV) exhibited behavioral fever, increasing their preferred temperature and upregulating pro-inflammatory cytokines (IL-1β, IL-6, TNF-α) [7]. Elevated body temperature during fever can also directly impair pathogen survival [6,7,18]. For example, zebrafish expressed sustaining behavioral fever for seven days effectively suppressed viral replication and prevented clinical symptoms [8]. These findings align with aquaculture practices where controlled thermal elevation is used to enhance disease resistance [19]. Given its broad immunological benefits, behavioral fever represents a promising, eco-friendly strategy for managing fish diseases [20].

The lined seahorse (*Hippocampus erectus*), valued both in traditional Chinese medicine and the ornamental fish trade, has been successfully cultured in captivity in China to resolve the conflict between ongoing commercial demand and the species’ precipitous decline in wild populations [21,22,23,24,25]. As unofficially announced, the annual cultured lined seahorses in dried form have reached more than 60.0 t in China since 2014 [24]. This species inhabits temperate waters with a natural temperature range of 20–29 °C [26,27], while optimal growth in aquaculture occurs at 25–26 °C [21,23,28,29]. Despite significant advances in commercial cultivation, disease outbreaks, particularly enteritis, remain a major challenge [30,31]. Enteritis can cause mortality rates up to 80% within 3–5 days of symptom onset, characterized by white pus around the anal opening and abdominal swelling [31]. Current disease management strategies include antibiotic treatment, environmental optimization, high-quality nutrition, and regular quarantine protocols [32,33,34]. Beyond conventional disease control measures, behavioral fever may offer an environmentally sustainable alternative by enabling seahorses to self-regulate their immune response, potentially reducing reliance on pharmaceutical treatments.

Based on the conserved nature of behavioral fever in ectotherms, we hypothesized, that *Vibrio harveyi* infection would induce behavioral fever in lined seahorses (*Hippocampus erectus*) and enhance their immune response through thermoregulatory behavior, as observed in other fish species. To evaluate this hypothesis, we designed a controlled experiment with two thermal regimes: constant temperature (25 °C) control group, and thermal gradient (19–31 °C) experimental group. We intraperitoneally injected seahorses with *V. harveyi* and allowed seahorse access to either a constant temperature environment, or a thermal gradient tank allowing free behavioral thermoregulation. Using integrated behavioral, physiological, and biochemical approaches, we quantified movement patterns and thermal preference, measured plasma cytokine levels (PGE2, IL-1β, IL-6, and TNF-α), and analyzed immune-related gene expression (*tnf-α*, *il-6*, *ifn-g*, *il-10*) in kidney tissue.

## 2. Materials and Methods

### 2.1. Experimental Seahorses

Four-month-old lined seahorses with a mean body weight of 2.99 ± 0.61 g (mean ± SD) and body height of 9.63 ± 0.61 cm were bred and obtained from the Qionghai Research Center of the East China Sea Fisheries Research Institute (Hainan, China). The seahorses were maintained in large tanks (4 × 3 × 1 m) with a central filtration system, ultraviolet sterilization unit, and plastic ropes holdfasts provided as attachment substrates. Culture environmental parameters were set as 32.0 ± 0.5‰ salinity, temperature 25.0 ± 0.5 °C, 800–1000 l× light intensity, and 13 h L:11 h D natural photoperiod. The seahorses were fed with frozen *Neomysis awatschensis* (harvested from Wudi County, Shandong Province, China) twice daily (09:00 and 16:00) at 15% wet body weight per day. The feces and uneaten food in the tanks were siphoned out 1 h post-feeding, and half of the total water was exchanged daily. All experimental procedures were approved by the Institutional Animal Care and Use Committee (IACUC #160413) of the Chinese Academy of Sciences and conducted in accordance with their ethical guidelines.

### 2.2. Thermal Gradient Experiment Setup

**Figure 1:**
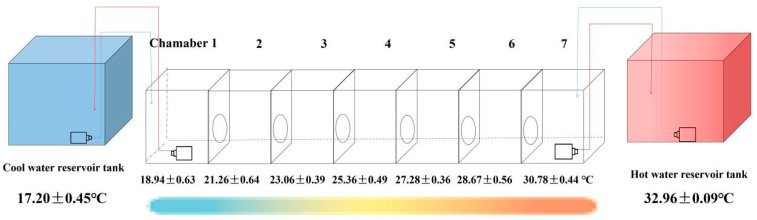
Thermal gradient tank setup. Polypropylene tank (250 (L) × 25 (W) × 25 (H) cm, 15 cm water depth) was divided into seven equal interconnected chambers (Chamber 1–7) by 2 cm thick foam partitions. Each board had a 12 cm diameter circular opening (4 cm above tank bottom) to allow seahorses inter-chamber access. The temperature control system was created by a cold terminus (Chamber 1) maintained at 17.20 ± 0.45 °C using a recirculating water chiller, and a warm terminus (Chamber 7) maintained at 32.96 ± 0.09 °C using dual 1000 W heaters, connected to a separate 60 × 45 × 45 cm reservoir tank, respectively. The recirculation was achieved via 2 W pumps connecting terminal chambers to respective reservoirs. Central airstones in each chamber mixed water to ensure homogeneous temperatures. The established thermal gradient exhibited the following stable profile across experimental trials from Chamber 1 to 7: 18.94 °C, 21.26 °C, 23.06 °C, 25.36 °C, 27.28 °C, 28.64 °C, 30.78 °C during the experiment.

The thermal gradient apparatus (Figure 1) was modified from the design described by Boltaña et al. [7,8]. The system comprising a main experimental tank and the temperature control system. The main experimental tank was a polypropylene tank with 250 (L) × 25 (W) × 25 (H) cm and 15 cm water depth. The tank was divided into seven equal interconnected chambers (Chamber 1–7) by 2 cm thick foam partitions. Each board had a 12 cm diameter circular opening (4 cm above tank bottom) to allow seahorses inter-chamber access. The temperature control system was created by a cold terminus (Chamber 1) maintained at 17.20 ± 0.45 °C using a recirculating water chiller (RESUN, Shenzhen, China), and a warm terminus (Chamber 7) maintained at 32.96 ± 0.09 °C using dual 1000 W heaters (JEBO, Zhongshan, Guangdong, China), connected to a separate 60 × 45 × 45 cm reservoir tank, respectively. The recirculation was achieved via 2 W pumps (Shenzhen, China) connecting terminal chambers to respective reservoirs. Central airstones in each chamber mixed water to ensure homogeneous temperatures. The established thermal gradient exhibited the following stable profile across experimental trials: 18.94 ± 0.63 °C (Chamber 1), 21.26 ± 0.64 °C (Chamber 2), 23.06 ± 0.39 °C (Chamber 3), 25.36 ± 0.49 °C (Chamber 4), 27.28 ± 0.36 °C (Chamber 5), 28.64 ± 0.56 °C (Chamber 6), and 30.78 ± 0.44 °C (Chamber 7). During the experiment, continuous temperature monitoring, uniform water flow between compartments, and daily verification of gradient stability were measured to control the temperature gradient quality.

### 2.3. Vibrio Harveyi Challenge and Behavioral Fever Assay

**Figure 2:**
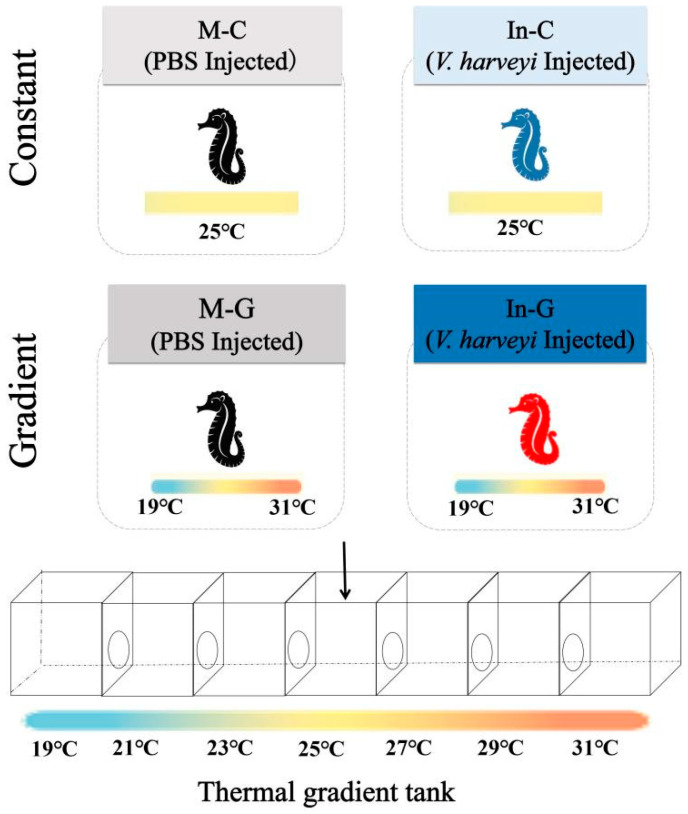
Schematic diagram of behavioral fever in the lined seahorses. In general, four experimental groups were set: mock in a constant temperature tank (M-C); mock in a thermal gradient tank (M-G); *V. harveyi* infection in a constant temperature (In-C); and *V. harveyi* infection in a thermal gradient tank (In-G). The constant tank has the constant temperature tanks with restricted thermal range (25 ± 0.2 °C); the thermal gradient tank was set as the gradient temperature with wide thermal range (25.0 ± 6.0 °C).

In vivo *V. harveyi* infection protocol aligned with established methodology from Lin et al. [31] as follows. Generally, the *V. harveyi* strain stocked in the lab [35] was freshly cultivated and resuspended in sterile 1× Phosphate-Buffered Saline (PBS, Solarbio, Beijing, China) to a final concentration of 2.0 × 10^8^ CFU/mL in prior. All injectable solutions underwent sterilization through autoclaving at 121 for 20 min and syringes were replaced between individuals to ensure the sterility. Prior to injection, experimental seahorses were anesthetized with 0.035% MS-222 (Sigma-Aldrich, Shanghai, China) to minimize solution leakage contamination caused by struggling. Generally, 160 seahorses were fasted for 24 h prior to injection and randomly divided into two main groups: infected group (In) and mock group (M), which received 50 μL bacterial suspension (1.0 × 10^7^ cfu/fish) and sterile 1 × PBS, respectively. All injections were performed intraperitoneally using 30 G needles. Each main group was further divided into constant temperature (C, 25.0 ± 0.2 °C) and thermal gradient (G, 25.0 ± 6.0 °C gradient range) treatments, subsequently, resulting in four experimental groups (N = 40 per group) (Figure 2): (i) M-C: mock-infected in constant temperature, (ii) M-G: mock-infected in thermal gradient; (iii) In-C: infected in constant temperature; (iv) In-G: infected in thermal gradient. Each experimental group consisted of 4 independent replicates (10 fish per trail). No feeding occurred during trials to prevent behavioral interference. All other husbandry conditions maintained as pre-challenge.

### 2.4. Behavioral Monitoring and Sample Collection

To monitor the seahorses’ movement across chambers in real time, two EZVIZ surveillance cameras (Hangzhou, China) were mounted on customer-built wood frames positioned above each tank. Seahorse movements were continuously recorded for 36 h under a natural photoperiod (13 L:11 D), with chamber temperatures monitored using calibrated digital thermometers (0.1 °C resolution). At the start of each trial, 10 seahorses were restricted in Chamber 4 (25.36 ± 0.49 °C) at 06:30. Following a 30 min acclimation period, the restriction was removed, allowing free movement, and recording commenced at 07:00 (Day 1) until 19:00 (Day 2) for a 36 h observation period for each test trail. Seahorse positions were manually quantified at 15 min intervals (144 time points total), with individuals counted in a chamber once their head crossed the partition plane. Throughout the experiment, clinical signs of *V. harveyi* infection, including abdominal distension, anal pus discharge, and impaired swimming, were documented for each individual. Samples (blood, kidney, and whole brain) were collected from three experimental groups, namely control (M-C, mock-infected seahorse randomly selected from constant temperature), no fever (In-C, infected seahorse randomly selected from constant temperature), and fever (In-G, infected seahorses randomly selected from Chamber 7 in gradient temperature).

### 2.5. Plasma Analysis by Enzyme-Linked Immunosorbent Assay (ELISA)

Seahorses were anesthetized for 2 min in 0.035% MS-222 solution (Sigma-Aldrich, Shanghai, China). Blood was sampled from the caudal artery via tail tip excision. The tail was immediately immersed in 400 μL anticoagulant solution (composition of 0.48% citric acid, 1.32% sodium citrate, 1.47% glucose, Solarbio, Beijing, China), allowing the blood to spontaneously mix with anticoagulant for 2 min. Afterwards, the blood samples were stored at 4 °C for 10 min, and then centrifuged at 840× *g* for 10 min at 4 °C. Plasma supernatant was collected and stored at −80 °C until analyses. The plasma protein concentration was quantified by a Total Protein Quantitative Assay Kit (coomassie brilliant blue, Nanjing Jiancheng Bioengineering Institute, Nanjing, China). Then, PGE2, interleukin (IL)-1β, IL-6, and tumor necrosis factor (TNF)-α concentrations of the plasma were determined using the commercial fish-specific ELISA kits (Nanjing Jiancheng Bioengineering Institute, Nanjing, China), following the manufacturer’s instructions.

### 2.6. Gene Expression Analysis by Quantitative Real-Time PCR (qRT-PCR)

Total RNA was isolated from kidney tissue using TRIzol reagent (Invitrogen, Waltham, MA, USA). Then, genomic DNA removal and first-strand cDNA synthesis were performed using PrimeScript^TM^ RT Reagent Kit with gDNA Eraser (TaKaRa, Beijing, China). For qPCR optimization, amplification efficiency (0.9–1.05) was validated using serial 4-fold cDNA dilutions, and all reactions included triplicate technical replicates were performed in 96-well plates on a Roche LightCycler 480 System (Basel, Switzerland). 10 μL total volume, contained 5 μL Green I Master, 1 μL of each forward and reverse primer (1 nM final concentration), 2 μL of cDNA, and 1 μL of ddH_2_O. Cycling conditions were set for 10 min at 95 °C, followed by 40 cycles of 10 s at 95 °C, 10 s at 60 °C and 8 s at 72 °C. The partial cDNA sequences of lined seahorses were obtained from the seahorse genome database [35], and gene-specific primers of *tnf-α*, *il-1β*, *il-6*, *ifn-g*, *il-2*, *il-10*, *tlr5*, and *pis* in the kidney, with *β-actin* serving as an endogenous reference gene, were designed (Table 1). The 2^−ΔΔCT^ method [36] was calculated to analyze the relative expression level.

### 2.7. Statistical Analysis

The number of seahorses in each chamber was manually counted by a single observer to minimize bias. Behavioral fever analysis was conducted using a generalized linear model (GLM) with Wald tests for pairwise comparisons between groups, applying the Bonferroni method to adjust the significance level [7]. The total number of seahorse occurrences across 144 experimental units was modeled by chamber location, vibrio challenge, and their interaction functions as independent variables. Prior to statistical analysis, the normality of physiological and biochemical parameters was assessed using Shapiro–Wilk’s W-test, while homogeneity of variance was evaluated with Levene’s test. Data meeting assumptions were analyzed by one-way ANOVA. For significant ANOVA results, post hoc comparisons were performed using Tukey’s HSD test. All data were analyzed in SPSS 19.0 (IBM Corp., Armont, NY, USA). Figures were generated using GraphPad PRISM v6.0 (GraphPad Software, Inc., San Diego, CA, USA).

## 3. Results

### 3.1. Thermoregulatory Behavior in V. harveyi Challenged Seahorse

**Figure 3:**
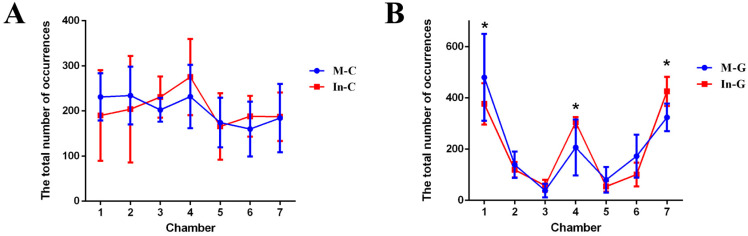
The observation of behavioral fever in mock and *V. harveyi*-infected lined seahorses in each chamber under the constant temperature (**A**) or thermal gradient temperature (**B**) tanks. (**A**) The total number of occurrences for the mock seahorses (M-C) had no significant difference with *V. harveyi*-infected seahorses (In-C) in each chamber under constant temperature. (**B**) A significantly higher number of occurrences for the infected seahorses were observed in the higher temperature Chamber 7 (31 °C) than the mock seahorses under the thermal gradient temperature condition (25 °C), indicating infection onset the behavioral fever of the lined seahorses to move to a warmer place. (N = 10, four replicates, the Wald test, * indicates *p* < 0.05).

To determine whether seahorses exhibit behavioral fever in response to *Vibrio harveyi* infection, we housed them in multi-chamber tanks under either constant or gradient thermal conditions post-infection and recorded their distribution across chambers over time. Under constant temperature conditions, Wald test results indicated no significant difference (*p* > 0.05) in the total number of occurrences between infected (In-C) and mock (M-C) seahorses in any chamber (Figure 3A), ranging from 165.75 to 275.25. Specifically, the total frequencies in Chamber 7 (25 °C) were 184.25 (mock) and 187.25 (infected), further supporting this conclusion. In contrast, under thermal gradient conditions (Figure 3B), both mock and infected seahorses aggregated preferentially in the two end chambers. Notably, in the warmest chamber (Chamber 7, 31 °C), infected seahorses (In-G) exhibited a significantly higher number of occurrences (425.50) compared to mock individuals (M-G, 324.25; *p* = 0.019, Wald test). Additionally, the total number of occurrences of the infected seahorses (In-G) significantly reduced in Chamber 1 (19 °C, 376.50 and 480.50 in the infected and mock group, respectively, *p* = 0.017, Wald test) and significantly increased in Chamber 4 (25 °C, 305.25 and 206.50 in the infected and mock group, respectively, *p* = 0.023, Wald test) compared to the mock seahorses, while no significant differences were observed in the rest—Chamber 2, 3, 5, and 6 (*p* > 0.05). These findings demonstrate that the increased occupancy of warmer chambers by infected seahorses was not due to inherent chamber preference but rather the onset of behavioral fever.

### 3.2. The Onset of Behavioral Fever in Seahorse

**Figure 4:**
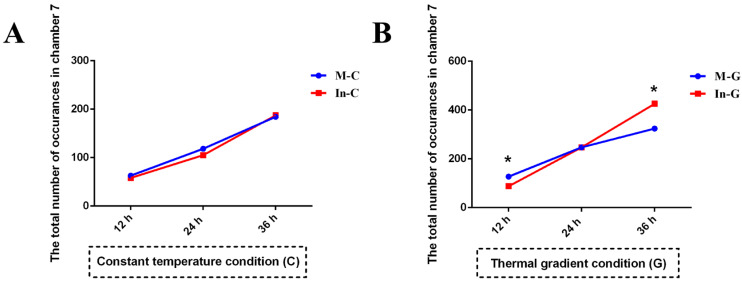
The total number of the mock and infected seahorses in the warmest Chamber 7 under two thermal conditions of constant temperature (**A**) and thermal gradient (**B**) over time (N = 10, four replicates, the Wald test, * indicates *p* < 0.05).

To determine the onset time of behavioral fever, we analyzed temporal changes in the distribution number of seahorses in Chamber 7 (the warmest chamber) under both constant and gradient thermal conditions. Under constant temperature, no significant differences (*p* > 0.05) were observed between mock and infected seahorses at any time point (12 h, 24 h, or 36 h; Figure 4A). In contrast, under thermal gradient conditions, the distribution patterns differed markedly over time. At 12 h, infected seahorses exhibited significantly lower occupancy in Chamber 7 compared to mock individuals (87.50 vs. 127.00 occurrences; *p* < 0.0001, Wald test). At 24 h, no significant difference was detected (247.25 occurrences for both groups; *p* > 0.05). By 36 h, infected seahorses showed significantly higher occupancy (425.50 vs. 324.25 occurrences; *p* = 0.019, as in Figure 4B). This kinetic migration pattern suggests that behavioral fever in infected seahorses develops between 24 h and 36 h post-infection. Detailed chamber-wise observations recorded at 15 min intervals over 36 h are provided in Supplementary Table S1.

### 3.3. Effect of Behavioral Fever on Immune Physiology

**Figure 5:**
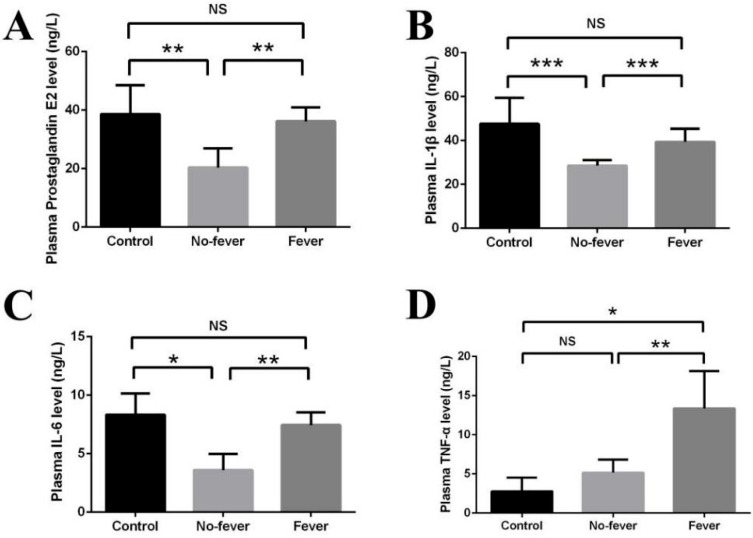
The released plasma pro-inflammatory cytokines in response to behavioral fever. (**A**) Plasma PGE2 level; (**B**) Plasma IL-1β; (**C**) Plasma IL-6; (**D**) Plasma TNF-α. Control: mock in a constant temperature (M-C). No-fever: *V. harveyi* infection in a constant temperature (In-C). Fever: *V. harveyi* infection in Chamber 7 with a temperature gradient (In-G). (N = 5, one-way ANOVA, NS indicates *p* > 0.05, * indicates *p* < 0.05, ** indicates *p* < 0.01, *** indicates *p* < 0.001).

The immunological results are presented in Figure 5. Plasma levels of inflammatory cytokines (PGE2, IL-1β, and IL-6) were significantly elevated (*p* < 0.05) in *V. harveyi*-challenged seahorses under thermal gradient conditions (fever group) compared to those maintained at constant temperature (no fever group). These cytokine levels returned to baseline (control group) by the end of the observation period (*p* > 0.05). In contrast, TNF-α concentrations remained significantly higher in the fever group compared to both the no fever and control groups (*p* < 0.05).

### 3.4. Effect of Behavioral Fever on Immune Gene Expression

**Figure 6:**
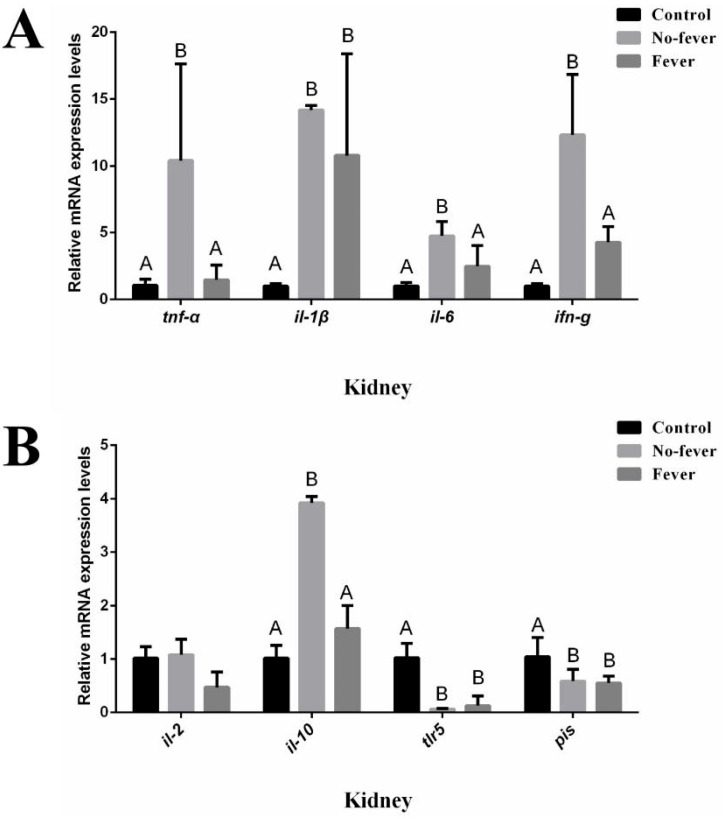
The expression of immune related genes in kidney during behavioral fever. Relative mRNA expression levels of (**A**) *tnf-α*, *il-1β*, *il-6*, and *ifn-g*; (**B**) il-2, *il-10*, *tlr5*, and *pis*. Control: mock in a constant temperature (M-C). No-fever: *V. harveyi* infection in a constant temperature (In-C). Fever: *V. harveyi* infection in Chamber 7 with a temperature gradient (In-G). (N = 3, one-way ANOVA, different capital letter indicates *p* < 0.05).

As demonstrated in Figure 6, kidney tissues from fever seahorses exhibited significantly higher mRNA expression levels of *tnf-α*, *il-6*, *ifn-g*, and *il-10* compared to the no-fever group (*p* < 0.05). When compared to unchallenged controls, *il-1β* expression was significantly elevated in both fever and no-fever groups, while *tlr5* and *pis* showed significantly lower expression (*p* < 0.05). No significant differences in *il-2* transcription were observed among groups (*p* > 0.05).

## 4. Discussion

Thermal stratification in natural aquatic environments enables fish to behaviorally thermoregulate through vertical or horizontal movement [37]. However, intensive aquaculture systems typically maintain commercially important species like seahorses at constant temperatures optimized for growth rather than health. For instance, lined seahorses (*Hippocampus erectus*) are conventionally cultured at 25–26 °C to maximize growth performance [28,29,33]. While behavioral fever represents a well-documented anti-pathogen response in fish [4,8], its potential application in aquaculture disease prevention remains largely unexplored. Our study provides the first evidence of behavioral fever in lined seahorses, suggesting this natural immune mechanism could be strategically employed in aquaculture to enhance disease resistance.

We observed that bacterial infection significantly increased the thermal preference of lined seahorses, with marked migration from 25 °C to 30 °C chambers. This was further supported by there being no significant difference in the occurrences between infected and mock seahorses in any chamber of the constant tank, suggesting that infection alone did not alter chamber preference. This thermoregulatory behavior represents a consistent febrile response, comparable to temperature elevations documented in rainbow trout (2.5 °C) [17], zebrafish (1.5–2.5 °C) [15], and Atlantic salmon (2.5–5.5 °C) [6]. The behavioral fever developed between 24 and 36 h post-infection (hpi), consistent with interspecies variation where Atlantic salmon exhibit fever at 24 hpi [6,7], rainbow trout at 16–18 hpi [17], and zebrafish at 12 hpi [8]. These temporal differences likely reflect species-specific physiological responses and pathogen characteristics.

At the physiological level, we found significantly elevated plasma PGE2 in febrile seahorses, consistent with its established role as an endogenous pyrogen in ectotherms [3,8]. The cytokine-mediated regulation of febrile responses appears evolutionarily conserved, with elevated IL-1β, IL-6, and TNF-α levels in febrile seahorses mirroring patterns observed in Atlantic salmon [7] and rainbow trout [17]. Notably, TNF-α had been specifically implicated in common carp behavioral fever [17], while multiple cytokines (IL-1β, IL-6, TNF-α, and interferons) serve as endogenous pyrogens in endotherms [9,10].

Our data demonstrate that behavioral fever enables lined seahorses to restore immune homeostasis, with febrile individuals achieving physiological parameters comparable to unchallenged controls. This suggests active thermal preference may synergistically enhance immune function. The observed inverse relationship between kidney gene expression and plasma cytokine profiles may reflect differential regulation, as febrile seahorses achieved cytokine homeostasis through behavioral thermoregulation (resulting in downregulated mRNA transcription), while non-febrile individuals maintained elevated gene expression due to persistent immune activation. These findings aligned with Boltaña et al. demonstration of epigenetic modifications remodeling immune-related gene expression in febrile Atlantic salmon, and confirm behavioral fever’s protective benefits, including reduced mortality and an absence of clinical infection signs [3,6].

Interestingly, we also observed increased seahorse movement to colder chambers (19 °C). While stress-induced hypothermia has been documented in fish [38], and our previous work showed *V. harveyi* infection induces both immune and stress responses in lined seahorses [35], the underlying mechanisms remain unclear. Similar to zebrafish that frequently visit chambers above and below their optimal temperature range (26–28 °C) [20], the thermal preferences of infected seahorses may reflect complex interactions between fever response and stress modulation. This warrants further investigation to distinguish between stress-induced hypothermia and active thermal preference.

From an aquaculture perspective, thermal choice under appropriate conditions can successfully orchestrate immune responses and growth [6,7,8]. The growing recognition of adaptive thermal choice as a welfare indicator [20,39] is exemplified by common carp that bask to elevate body temperature by ~4 °C, enhancing growth [40]. Whereas forced thermal stress without pathogen-induced behavioral choice may negatively impact oxidative stress responses [41,42], innate immunity, and glucocorticoid responses [43,44]. For instance, acute thermal stress at 30 °C affects respiration rate, Hsp gene expression, and appetite in healthy lined seahorses [25]. In seahorse aquaculture practice, upon observation of clinical signs [31], or aberrant behavior including failure to anchor via prehensile tails, immediate manual translocation of affected individuals from the primary culture pond to auxiliary tanks with elevated water temperatures (e.g., 3–5 °C above ambient) may be implemented as a mitigation protocol.

The ecological implications of these findings are significant. While seahorse migration is typically attributed to feeding, habitat preference, reproduction, and anthropogenic factors [25], our study identifies pathogen infection as an additional behavioral driver through behavioral fever induction. Importantly, this response is specifically triggered by exogenous pyrogens (e.g., bacteria or viruses) [11], distinct from stress-induced emotional fever [38]. Currently, most research focuses on the immune benefit of instant episodes of behavioral fever, its long-term impact on seahorse health and production requires further investigation. Future studies should examine the fitness consequences of behavioral fever in natural populations, the interaction between fever response and stress modulation, and practical applications for improving seahorse aquaculture through strategic thermal management.

## 5. Conclusions

This study comprehensively investigated the thermal preference behavior of lined seahorses following *V. harveyi* infection and elucidated the synergistic relationship between behavioral fever and immune response. Our findings demonstrate that infected seahorses actively migrate to warmer environments (31 °C) in thermal gradient tanks within 36 h post-infection, exhibiting a clear behavioral fever response. This thermoregulatory behavior facilitated an effective immune response, as evidenced by significant changes in cytokine profiles and immune-related gene expression patterns. Critically, we reported the importance of creating a natural environment for aquaculture animals to fully express a normal behavior, therefore reinforcing the efficacy of immune defense. We also propose accordingly elevating temperature as a viable alternative for ecological control diseases in the lined seahorse aquaculture.

## Figures and Tables

**Table 1:** mRNA primer sequences used for qRT-PCR analysis.

Gene	Primer 5′-3′
*tnf-α*	F:TGCCAGCACAGCAGTTCG
	R:TTGCCGCCGTCCGTTT
*il-1β*	F:CAAGCACAACCCTAACCACA
	R:GAGACCTCCAGGCTCAATCC
*il-6*	F:CTCCTCCAACTTCAGCAAGG
	R:TGTGACCTCCTCTGGCTTTT
*ifn-g*	F:CGCCTACTTGTGTCTGTGTC
	R:GCAGCTCCTCGTACGTCT
*il-2*	F:ACTCGCTCCACTGGCTTCC
	R:CCCTGCTCTGTGCTCCTCA
*il-10*	F:GAGGACACGAGGGACTTGAA
	R:AGCTCGCCCATAGCTTTGTA
*tlr5*	F:ACGTTACATATTTGGACATTCGG
	R:TCAAGGTCAAACTGAGCAAATGG
*pis*	F:CTGGTCACGCTCTTCCTG
	R:TGTCGGTTCTCCCAAGC
*β-actin*	F:ACCATGTACCCTGGCATTG
	R:TCATACTCCTGCTTGCTGAT

*tnf*, tumor necrosis factor; *il*, interleukin; *ifn*, interferon; *tlr*, toll-like receptor; *pis*, piscidin.

## Data Availability

The data presented in this study are available on request from the corresponding author due to ethical, legal, or privacy restrictions.

## Supplementary Materials

The following supporting information can be downloaded at https://www.mdpi.com/article/10.3390/ani15111509/s1, Table S1: Distribution of seahorses in each group across different chambers over time.
